# Fluctuation in the diversity of mayflies (Insecta, Ephemerida) as documented in the fossil record

**DOI:** 10.1038/s41598-023-42571-7

**Published:** 2023-09-25

**Authors:** Pavel Sroka, Roman J. Godunko, Jakub Prokop

**Affiliations:** 1 Biology Centre of the Czech Academy of Sciences, Institute of Entomology, Branišovská 31, 370 05 České Budějovice, Czech Republic; 2 Department of Invertebrate Zoology and Hydrobiology, University of Łodź, Banacha 12/16, 90237 Łodź, Poland; 3 Department of Zoology, Faculty of Science, Charles University, Viničná 7, 128 00 Prague 2, Czech Republic

**Keywords:** Entomology, Palaeontology, Speciation, Freshwater ecology

## Abstract

Due to their aquatic larvae, the evolution of mayflies is intricately tied to environmental changes affecting lakes and rivers. Despite a rich fossil record, little is known about the factors shaping the pattern of diversification of mayflies in deep time. We assemble an unprecedented dataset encompassing all fossil occurrences of mayflies and perform a Bayesian analysis to identify periods of increased origination or extinction. We provide strong evidence for a major extinction of mayflies in the mid-Cretaceous. This extinction and subsequent faunal turnover were probably connected with the rise of angiosperms. Their dominance caused increased nutrient input and changed the chemistry of the freshwater environments, a trend detrimental mainly to lacustrine insects. Mayflies underwent a habitat shift from hypotrophic lakes to running waters, where most of their diversity has been concentrated from the Late Cretaceous to the present.

## Introduction

Mayflies represent a very ancient group of insects, with stem-group representatives dating back to the Late Carboniferous, albeit these Pennsylvanian records are solely known from imagoes^1,2^. The putative mayfly larvae assigned to the Syntonopteroidea (Carboniferous and Permian) are considered doubtful^3,4^. Extant mayflies are well known for the extremely short life span of their winged stage – ranging from several hours to several days^5^ – and their dominant larval life stage (nymph) is exclusively aquatic.

During the long evolutionary history of mayflies, many profound changes in their freshwater ecosystems have taken place, resulting from changes in global temperature, altered climate, hydrological regime, or floral turnovers, such as the rise of aquatic macrophytes in the Triassic^6^ or the appearance of angiosperms in the Early Cretaceous^7^. Some of these deep environmental changes must have necessarily affected the origination and extinction of various mayfly lineages, as documented for other insects^8,9^. Relatively little attention has been paid to the timing and tempo of mayfly diversification, and its link with changes in the freshwater environment. An overview of the ecological factors affecting the diversification of aquatic insects was provided by Sinitshenkova^10,11^. As one of the general observations, it was noted that the Jurassic period and the Early Cretaceous are characterised by diverse and widespread lacustrine insect communities, whereas the Late Cretaceous assemblages were considerably impoverished.

Methodologies allowing the analysis of diversification and extinction patterns based on fossil data have recently been developed^12–14^. These methodologies rely on a Bayesian process-based model that incorporates temporal preservation biases. These approaches enable the identification of periods of high diversification or extinction and search for correlations with geological and environmental conditions^15^. Within aquatic insects, such a diversification analysis was recently conducted for stoneflies (Plecoptera)^16^. Mayflies represent a group with a similar ecology to stoneflies, yet they are more diversified in terms of ecomorphology and life strategies. They exhibit various feeding types (scrapers, filter feeders, predators, etc.), body shapes, and locomotion types (fish-like, flattened, burrowing, etc.), connected with their particular ecological niche^17,18^. Most of these life strategies are also documented in the fossil record. This diversity allows individual lineages to occupy various types of freshwater microhabitats, and it could have allowed the group as a whole to cope better with changing environmental conditions and made them less susceptible to mass extinction events.

Herein, we aim to determine if the pattern of diversification of mayflies followed a similar scenario as in stoneflies, or if differences in ecology caused a different scheme of evolution in mayflies. We also summarise a spatiotemporal distribution of the mayfly fossil record and provide an improved understanding of biases impeding possible interpretations. Finally, we clarify the role of environmental drivers in the major origination and extinction events experienced by mayflies during their evolutionary history.

## Results

### List of mayfly fossil occurrences

All the described fossil occurrences of mayflies attributable at least to the genus level are summarised in Supplementary Table 1. We provide detailed information concerning the age and number of occurrences for each species.

### Spatiotemporal distribution of mayfly fossils

**Figure 1:**
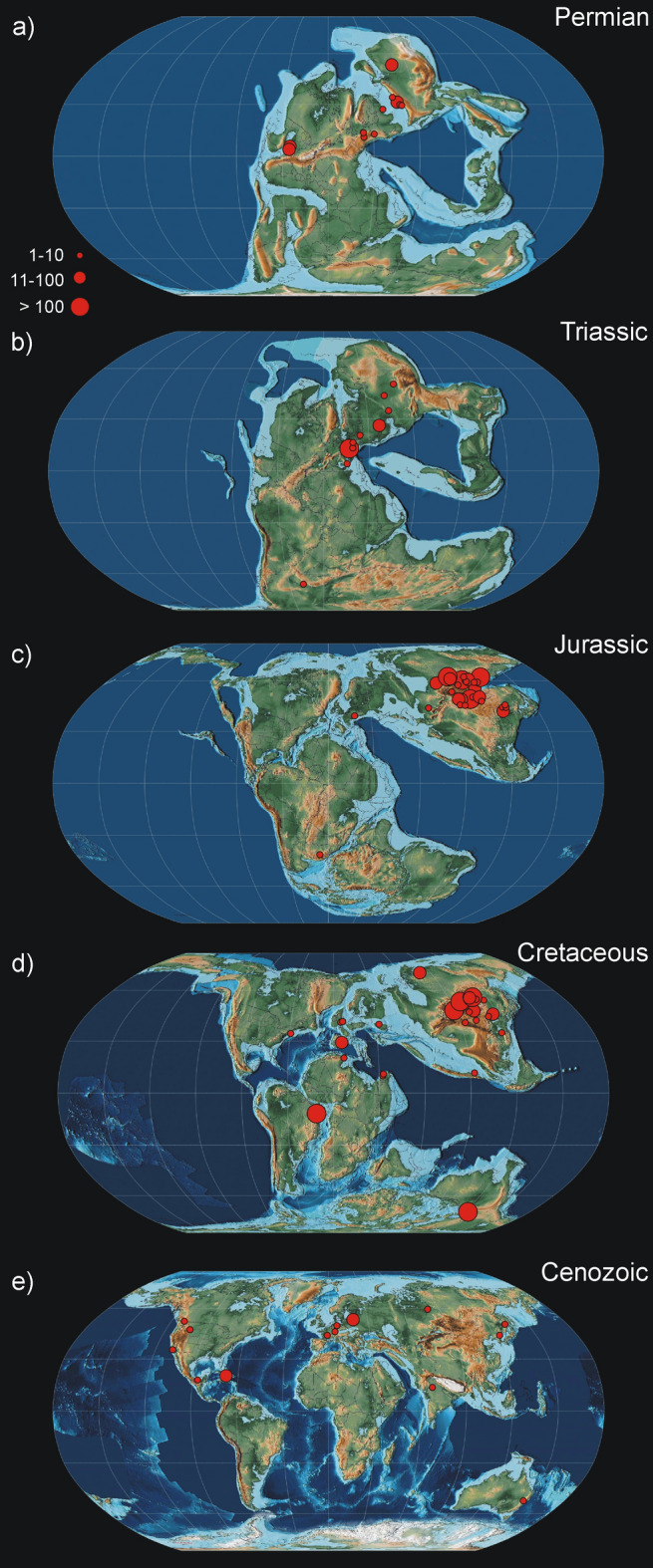
Palaeomaps with known occurrences of mayfly fossils through geologic time. Location of fossil sites is marked by red circles. The size of a circle indicates the richness of the locality (number of mayfly fossil occurrences). Maps generated using GPlates 2.3.0^19^ and raster images from Scotese^20^.

**Figure 2:**
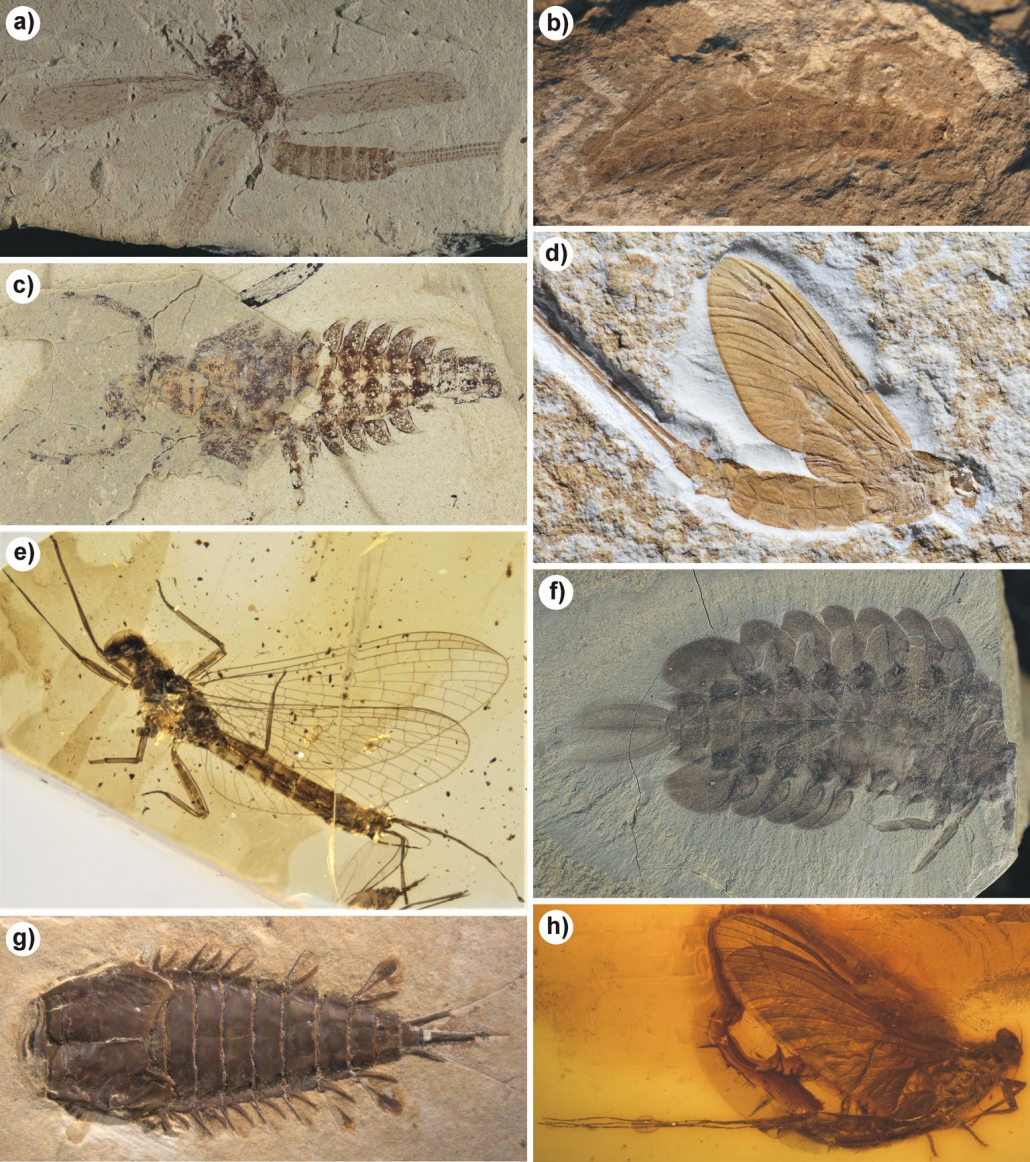
Mayfly fossils representing various taxa, periods and geographical locations. (**a**) *Misthodotes delicatulus*, Permian, Elmo, USA. (**b**) *Protereisma americana*, Permian, Midco, USA. (**c**) *Vogesonympha ludovici*, Triassic, Grès à Voltzia, France (holotype). (**d**) *Hexagenites cellulosus*, Jurassic, Solnhofen, Germany. (**e**) *Burmella paucovenosa*, Cretaceous, Burmese amber, Myanmar (holotype). (**f**) *Promirara cephalota*, Cretaceous, Koonwarra fossil beds, Australia (holotype). (**g**) *Cratohexagenites minor*, Cretaceous, Crato Formation, Brazil (holotype). (**h**) *Eurylophella viscata*, Eocene, Baltic amber, Poland (holotype).

**Figure 3:**
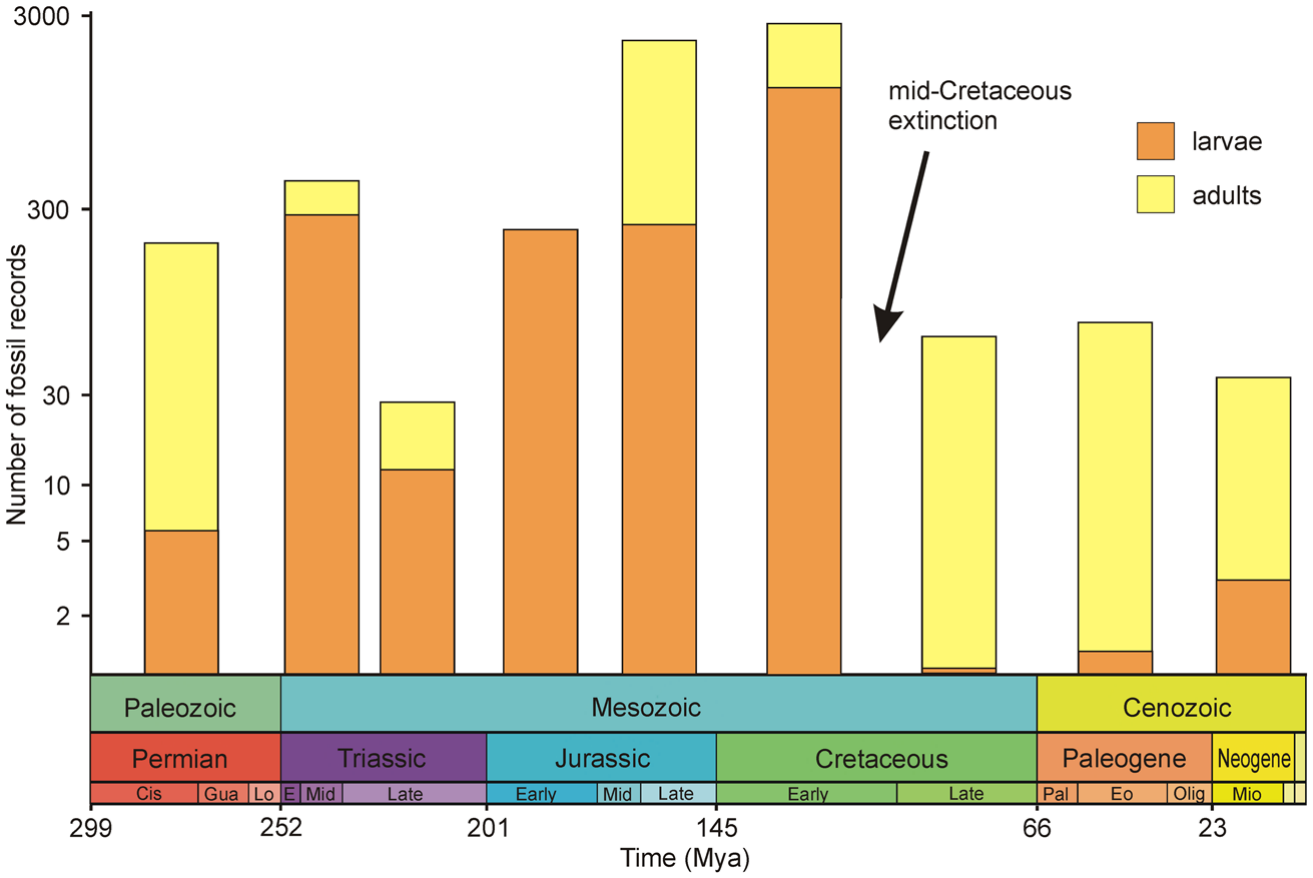
Richness of mayfly fossil record. Proportion of larvae and adults indicated with different colours. Y-axis in logarithmic scale. *Cis* Cisuralian, *E* Early, *Eo* Eocene, *Gua* Guadalupian, *Lo* Lopingian, *Mid* Middle, *Mio* Miocene, *Olig* Oligocene, *Pal* Paleocene. The occurrences were pooled for the following epochs: Early+Middle Triassic, Middle+Late Jurassic. The colour of each period in the chronostratigraphic scale follows that of the International Chronostratigraphic Chart (v2022/02).

The majority of Permian and Triassic localities are located in the Northern Hemisphere in the area of present-day Europe and Asia (Fig. 1a,b). From the Triassic, the vast majority of the material originates from just a single locality (Grès à Voltzia in France) and a few others less prolific such as Mallorca (Spain) or Madygen in Kyrgyzstan. For the Jurassic localities, there is a strong bias towards Asia, with nearly all mayfly occurrences found within Russia, Mongolia, and China (Fig. 1c). Only six occurrences in Solnhofen (Germany) and two in South Africa complement the mayfly record for the Jurassic. For the Early Cretaceous, mayfly occurrences are more evenly distributed throughout the globe and a significant amount of data from the Southern Hemisphere is recorded for the first time. Highly prolific localities are represented by the Crato Formation in Brazil and the Koonwarra fossil beds in Australia. Other rich localities also exist particularly in Asia (Fig. 1d). The amount of material sharply peaks at the end of the Jurassic and the beginning of the Cretaceous; multiple localities yielding rich material exist (Fig. 3). The most significant gap is the Late Cretaceous with only one compression/imprint fossil mayfly documented from this epoch: a larva attributable only to the order-level due to insufficient preservation of diagnostic characters^21^. All other occurrences of mayflies are limited to inclusions in amber from several deposits (Burmese/Myanmar, New Jersey, Lebanon, Taimyr). In the Cenozoic, most occurrences originate from amber deposits in the Northern Hemisphere (mainly Baltic and Dominican ambers) (Fig. 1e). Several examples of the mayfly fossils representing various periods and geographical locations are presented in Fig. 2.

When separately evaluating larvae and adults, the proportion of larvae is relatively comparable with the adults in the Permian, whereas larvae form the majority of occurrences from the Triassic to Early Cretaceous. From the Late Cretaceous onwards, adult occurrences predominate (Fig. 3). This is because the fossil record of mayflies, from the Cretaceous to the present, consists mostly of amber inclusions, which extremely rarely trapped aquatic stages^22^.

### Origination and extinction analysis

**Figure 4:**
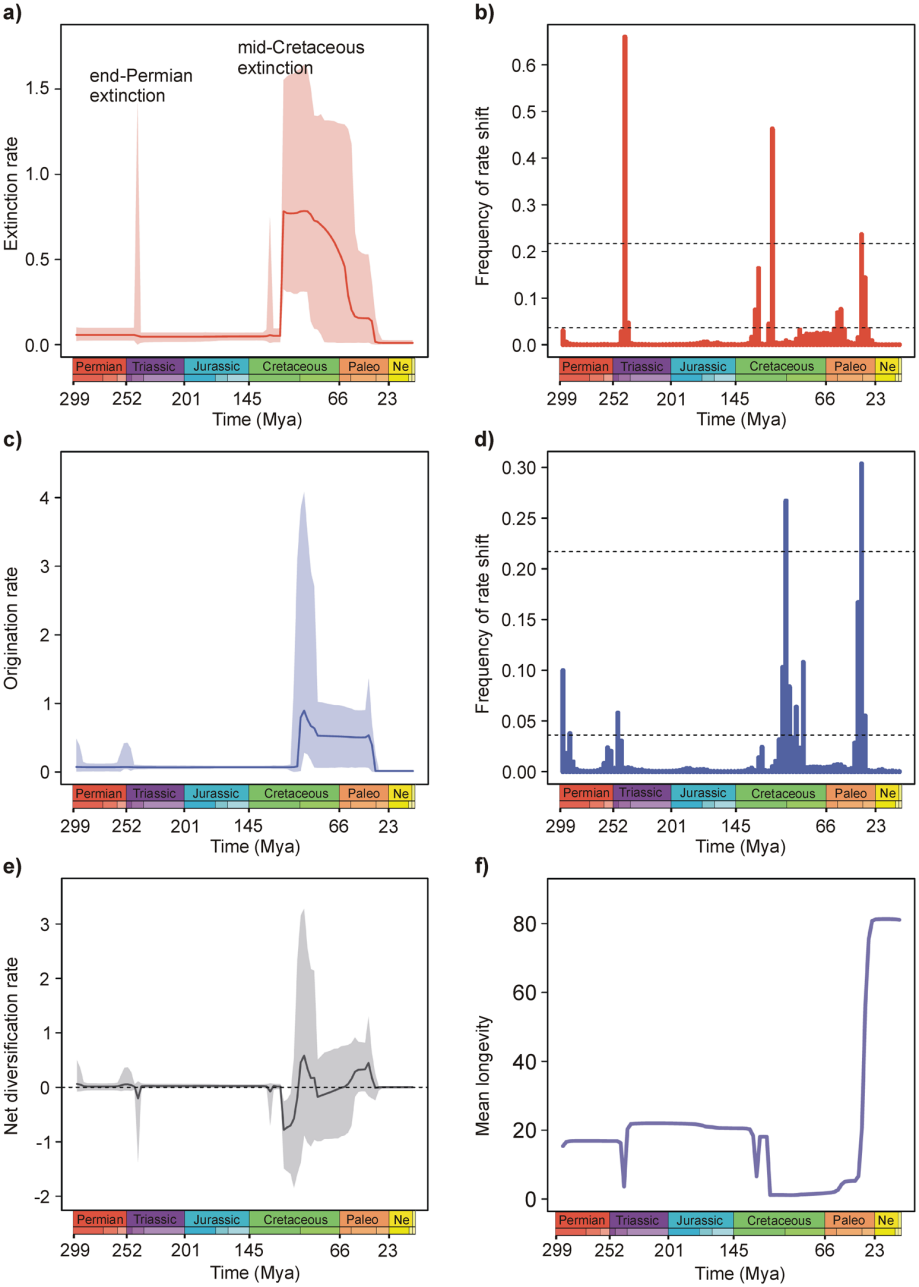
Diversification and diversity dynamics of mayflies in deep time. (**a**) Extinction rate. (**b**) Frequency of shifts in extinction rate. (**c**) Origination rate. (**d**) Frequency of shifts in origination rate. (**e**) Net diversification rate. (**f**) Longevity of lineages. The frequency of a sampled rate shift is computed within small time bins for origination and extinction rates ((**b)** and (**d**), respectively), with horizontal dashed lines indicating log-Bayes factors of 2 (bottom) and 6 (top). Sampling frequencies higher than log-Bayes factors = 6 indicate strong statistical support for a rate shift. The net diversification rates are obtained with the difference between origination and extinction rates (rates below 0 indicate declining diversity). Solid lines indicate mean posterior rates and the shaded areas show 95% credibility intervals. *Ne* Neogene, *Paleo* Paleogene. The colour of each period in the chronostratigraphic scale follows that of the International Chronostratigraphic Chart (v2022/02).

Our analysis revealed two distinct periods characterized by increased extinction rates in Ephemerida (Fig. 4a). The first period occurred around 242 million years ago and was marked by a rapid and relatively short burst of extinctions, coinciding with the Permian–Triassic extinction event. Following this event, the extinction rate declined and remained relatively stable until the late Early Cretaceous, around 116 million years ago.

However, at that point, the extinction rate sharply increased, reaching its maximum and persisting for approximately 24 million years. Subsequently, a period of decline in the extinction rate commenced, with the most significant drop occurring during the Eocene/Oligocene transition, approximately 35 to 30 million years ago.

The two mass extinction events (Permian-Triassic and late Early Cretaceous) correspond with the two most pronounced shifts in extinction rate found in our analysis (Fig. 4b).

The origination rate remained relatively stable until approximately 102 million years ago, after which a notable increase in origination occurred. This surge in origination closely followed the substantial rise in the extinction rate during the Cretaceous period (Fig. 4c). It indicates a significant turnover in the Ephemerida fauna following the initial major decline. The origination rate peaked around 96 million years ago and subsequently declined, with a significant drop observed in the late Eocene, around 36 million years ago (Fig. 4c).

Following the end-Permian mass extinction, the net diversification rate in Ephemerida experienced a temporary decline due to an increased extinction rate, while the origination rate remained relatively constant (Fig. 4e). The most significant decline occurred during the late Early Cretaceous, characterized by a sharp rise in the extinction rate, resulting in a decrease in net diversification. However, shortly after this decline, there was an upsurge in origination, leading to an increase in net diversification, reaching its peak during the Late Cretaceous, approximately 96 million years ago (Fig. 4e). Subsequently, there was another drop in net diversification, followed by a peak in the Eocene around 41 million years ago (Fig. 4e).

The timing of main periods of increased extinction and origination revealed by additional analyses without singletons or using BDMCMC algorithm is congruent with RJMCMC (Supplementary Fig. 1).

## Discussion

### Mass extinction events

Among the five most drastic extinction events in Earth’s history, called the Big Five^23,24^, we found an increase of mayfly extinction only in connection with the Permian–Triassic event, the most devastating mass extinction. Jouault et al.^16^ provided evidence that it had similarly drastically influenced stoneflies, although crises during the Permo–Triassic had a heterogeneous effect on other insect groups^25^. No significant peaks in mayfly extinction were detected during other events, including the end-Cretaceous mass extinction, aligning with previous findings that suggest insects were less affected by mass extinctions compared to larger organisms. The small size of insects, their ability to hide, and their resistant dormant stages like eggs likely contributed to their survival^9^. However, Labandeira^26^ identified three independent mass extinctions of insects, unrelated to mass extinctions in other taxa. Our results indicate that one of these events had a profound impact on the mayfly fauna, occurring in the late Early Cretaceous, specifically during the Albian and Aptian stages.

The Cretaceous Period (145–65 Mya) has usually been regarded as time of major reorganisation and modernisation of ecosystems. In the marine realm, these ecosystem changes are known collectively as the Mesozoic Marine Revolution^27^. Land-dwelling organisms experienced a similar event, the Cretaceous Terrestrial Revolution (KTR)^28^. The last of the Cretaceous revolutions was the Mesozoic Lacustrine Revolution (MLR), characterised by Buatois et al.^29^ as a series of major changes that took place in lacustrine environments during the mid-Mesozoic and is synchronous with an extinction event for aquatic insects^11,30^.

Our findings indicate that the mid-Cretaceous period aligns with a significant extinction event in mayflies, which began around 116 million years ago (Fig. 4a). This extinction was followed shortly by a peak in speciation, resulting in a turnover of the mayfly fauna. Similar turnover patterns were observed in stonefly fauna during the KTR, as demonstrated by Jouault et al.^16^. Remarkably, there is a striking resemblance between the diversification patterns of mayflies and stoneflies throughout the Mesozoic (fig. 2 in Jouault et al.^16^ and Fig. 4 in our study). Sinitshenkova^11^ provides several examples of other taxonomic groups, such as aquatic beetles, caddisflies, and aquatic flies, that also experienced drastic extinctions in lacustrine environments during the mid-Cretaceous. These findings suggest that profound changes in freshwater ecosystems had a significant impact on the structure of aquatic biota, affecting many components, including aquatic insects. The reasons behind these environmental changes, particularly their impact on aquatic insects, warrant further investigation.

### The rise of angiosperms

During the Jurassic and Early Cretaceous, most mayfly fossils come from oligotrophic low-saprobity lakes, which constituted a common type of habitat, being classified into several subtypes^31,32^. A rich mayfly fauna is reported from these oligotrophic lakes, whereas the existence of eutrophic water bodies was very limited^32^.

The oligotrophic nature of the pre-KTR lakes was probably chiefly maintained by the fact that the vegetation was mostly composed of gymnosperms. Gymnosperms could thrive at low nutrient supplies, but also maintained low nutrient availability in the soil, as a result of the kind of litter they produced^33^. The low availability of nutrients in the soil and leaf litter was probably responsible for the low saprobity of water bodies. There were alternative explanations, such as the hypothesis of Zherikin & Kalugina^31,34^ that the leaf litter of Mesozoic ginkgoleans and czekanowskialeans had strong bactericidal activity and was not decomposed, thus keeping those lakes hypotrophic and leaving enough oxygen for oxyphilic benthic insects. This hypothesis was challenged by Ponomarenko^35^, who argued that normal decomposition is proved for Mesozoic forest soils and also that lake sediments do not contain an undecomposed mass of foliage.

During the Mesozoic, the major biotic reorganisation was related to the rise of angiosperms (flowering plants). While the exact dating of their appearance is still debated^36,37^, knowledge from fossils attests the presence of angiosperms since the mid-Early Cretaceous Period^38^. Although molecular datings have raised the possibility that angiosperms may have existed much earlier (see summary of dating estimates in Barba-Montoya et al.^39^), there is no unequivocal evidence for pre-Cretaceous angiosperms^40^. Angiosperms progressively came to dominate the majority of terrestrial ecosystems^41,42^. The reasons why the angiosperms took over most ecosystems are still debated^33,43,44^. In any case, angiosperm plants exhibit several characteristics that affected the ecosystems they came to dominate, most importantly related to nutrient cycling and availability.

The evolution of high leaf vein density was likely a critical step enabling increased carbon assimilation^45^, particularly when coupled with other advances in resource acquisition by roots^43^. Thin, fast-growing roots, more common within angiosperms, can be superior for nutrient uptake relative to the thick, slow-growing roots of more ancient gymnosperms^46^. Moreover, the rapidly growing leaves of angiosperms tend to have short lifespans, which correlates with higher nitrogen levels in leaves, causing a general rise in nitrogen levels in soils arising from angiosperm productivity^7^. In general, the presence of angiosperms significantly increased the amount of nutrients in leaf litter and soil. Subsequently, these nutrients necessarily reached surface waters as runoff from precipitation.

In aquatic environments, an increased input of nutrients (in particular nitrogen and phosphorus) has many effects on the chemistry and biology of water bodies, such as elevation of pH, dissolved oxygen depletion, reduction of water clarity, and increased biomass of freshwater phytoplankton and periphyton^47^. For illustration, the consequences can be compared with the anthropogenic eutrophication caused by the excessive nutrient enrichment of today, recently identified as one of the drivers underlying ongoing arthropod population declines^48^. The consequences are much more pronounced in standing water bodies compared to rivers, due to greater flow in the latter, which causes nutrient transport rather than settlement^49^. Mayflies have been identified as one of the groups particularly sensitive to the negative effects of nutrient additions^48^ and we hypothesise that phenomenon was responsible for the pronounced increase of their extinction rate in the mid-Cretaceous.

### Larvae vs. adults in the fossil record

The process of the demise of mayflies in the lacustrine settings following the KTR can be demonstrated by a comparison of the fossil record of larvae and adults. To date, virtually no larvae have been found from the period following the late Early Cretaceous (Fig. 3). The fossil record of mayflies is then limited mostly to adults, often preserved as amber inclusions. Despite the continuous lack of fossils of larvae, mayflies as a whole did not decline all the time. Following the initial mass extinction connected with the KTR, the extinction rate decreased, being suppressed by the origination rate, resulting in diversification peaks in the Late Cretaceous and in the Eocene (Fig. 4e). However, this process did not coincide with an increased discovery of fossil larvae (Fig. 3). The scarcity of mayfly larvae fossils cannot be attributed to a lack of potentially suitable fossilisation sites, as several Late Cretaceous and Cenozoic localities with exquisitely preserved fossils of other insect groups exist^50^. Therefore, the question arises: why are there almost no fossils of mayfly larvae, not only shortly after the mid-Cretaceous extinction event but also throughout the 100-million-year period spanning the mid-Cretaceous to the present?

The circumstances of fossilisation differs significantly between different life stages. Mayfly larvae, being exclusively aquatic, tend to fossilize in situ within the sediment of their aquatic habitats. The majority of deposits for compression fossils are lakes or lagoons, with rheophilous fauna living more upstream preserved only as occasional allochthonous specimens, swept from the upstream river sections and extremely rarely found^34^. As a result, the fossil record of mayfly larvae is inherently biased towards taxa inhabiting still waters. Although mayflies also occupied running waters, their larvae are rarely documented in the fossil record.

Lentic taxa thrived in the oligotrophic lakes of the Jurassic and Early Cretaceous as demonstrated by the abundance and diversity of their fossils. Following the KTR, a large portion of mayfly diversity in the lacustrine habitats went extinct, whereas the taxa inhabiting running waters were much less affected. These riverine mayflies further diversified; their fauna evidenced as adults preserved in amber. Possibly some lacustrine lineages shifted to rivers, since living in oligotrophic lakes provided them with suitable preadaptations for life in well-oxygenated water bodies. Mid-Cretaceous insect fossil records indicate a decline in lacustrine diversity for various groups of aquatic insects, while their diversity in streams remained relatively high, as demonstrated by preserved specimens in amber^11^. Unlike these other groups, mayflies did not experience a subsequent diversification in standing water bodies. Instead, their primary diversity and abundance have remained concentrated in running waters since the middle of the Cretaceous until the present.

## Methods

### Dataset

The list of fossil occurrences was compiled based on online databases (http://www.fossilworks.org/, https://paleobiodb.org: last accessed 13 December 2022). The original dataset was supplemented with data acquired from the literature published until 31 December 2022 and our own investigation of individual collections. The table summarising the taxa included in our dataset, along with the numbers of occurrences is presented as Supplementary Table 1.

We restricted our analysis to Ephemerida^51,52^, containing the order Ephemeroptera and its close stem-group (Coxoplectoptera, Sinebranchia, and Permoplectoptera). We excluded Carboniferous members of the stem-group (Syntonopteroidea) due to their unclear phylogenetic affinities and the uncertain presence of aquatic larvae in this group. The final dataset contained 6345 records from 141 genera. We used only records identifiable to the genus level. We included data with reasonably reliable references in the published literature. Several taxa were excluded, mainly because of the doubtful status of the respective fossils (see Supplementary Table 1 for details). This dataset has limitations, since undescribed or even unsorted mayfly fossils are certainly present in some collections. We tried to make it as complete as possible by including additional records based on our unpublished data. Palaeomaps were generated using GPlates 2.3.0^19^ and raster images from Scotese^20^.

### Analysis

To estimate the temporal dynamics of origination and extinction, we used a Bayesian framework implemented in PyRate 3.0^12–14^. The input files were generated using R 4.2.1^53^ and the extract.ages function. We generated ten datasets with randomly resampled times between the minimum and maximum values for each fossil occurrence. To select the preservation model which fits the best with the data we used a maximum likelihood to test which among the non-homogeneous Poisson process (NHPP), the homogeneous Poisson process (HPP), or the time-variable Poisson process (TPP) best fits with the data^14^. The best model was identified as TPP (P ˂ 0.01). It assumes that preservation rates are constant within a predefined time frame, but can vary across time frames (in our case, geological epochs). A gamma model was added to account for heterogeneity in the preservation rate across lineages.

The resulting analysis was run to estimate origination and extinction rates through time. The number and temporal placement of shifts are estimated from the data using the RJMCMC algorithm (-A 4), since it is recommended for generally providing more accurate results compared to the alternative, BDMCMC algorithm^14^. We ran PyRate for 10 million MCMC iterations with a sampling frequency of 1000 and combined the posterior samples of the parameters from the 10 randomly replicated datasets. We excluded the first 10% of the samples as burn-in after inspecting the log files in Tracer 1.6. To check for the sensitivity of results, we ran two additional analyses, one using BDMCMC algorithm (A 2), and one with the RJMCMC and excluded singletons.

### Supplementary Information

Supplementary Figure 1.

Supplementary Table 1.

## Data Availability

All data needed to evaluate the conclusions in the paper are present in the paper and its supplementary information files, or from P.S. (pavel.sroka@centrum.cz) upon reasonable request.
