# Supplementary material for: Fluctuation in the diversity of mayflies (Insecta, Ephemerida) as documented in the fossil record
Source: Sci Rep. 2023 Sep 25;13:16052. doi: 10.1038/s41598-023-42571-7 (PMC10519997; doi:10.1038/s41598-023-42571-7)

**Supplementary Figure 1.** **Additional analyses of diversification and diversity dynamics of mayflies in deep time.** **a-f)** RJMCMC algorithm with excluded singletons, **g-j)** BDMCMC algorithm. **a, g)** Extinction rate. **b)** Frequency of shifts in extinction rate. **c, i)** Net diversification rate. **d, h)** Origination rate. **e)** Frequency of shifts in origination rate. **f, j)** Longevity of lineages. The colour of each period in the chronostratigraphic scale follows that of the International Chronostratigraphic Chart (v2022/02).


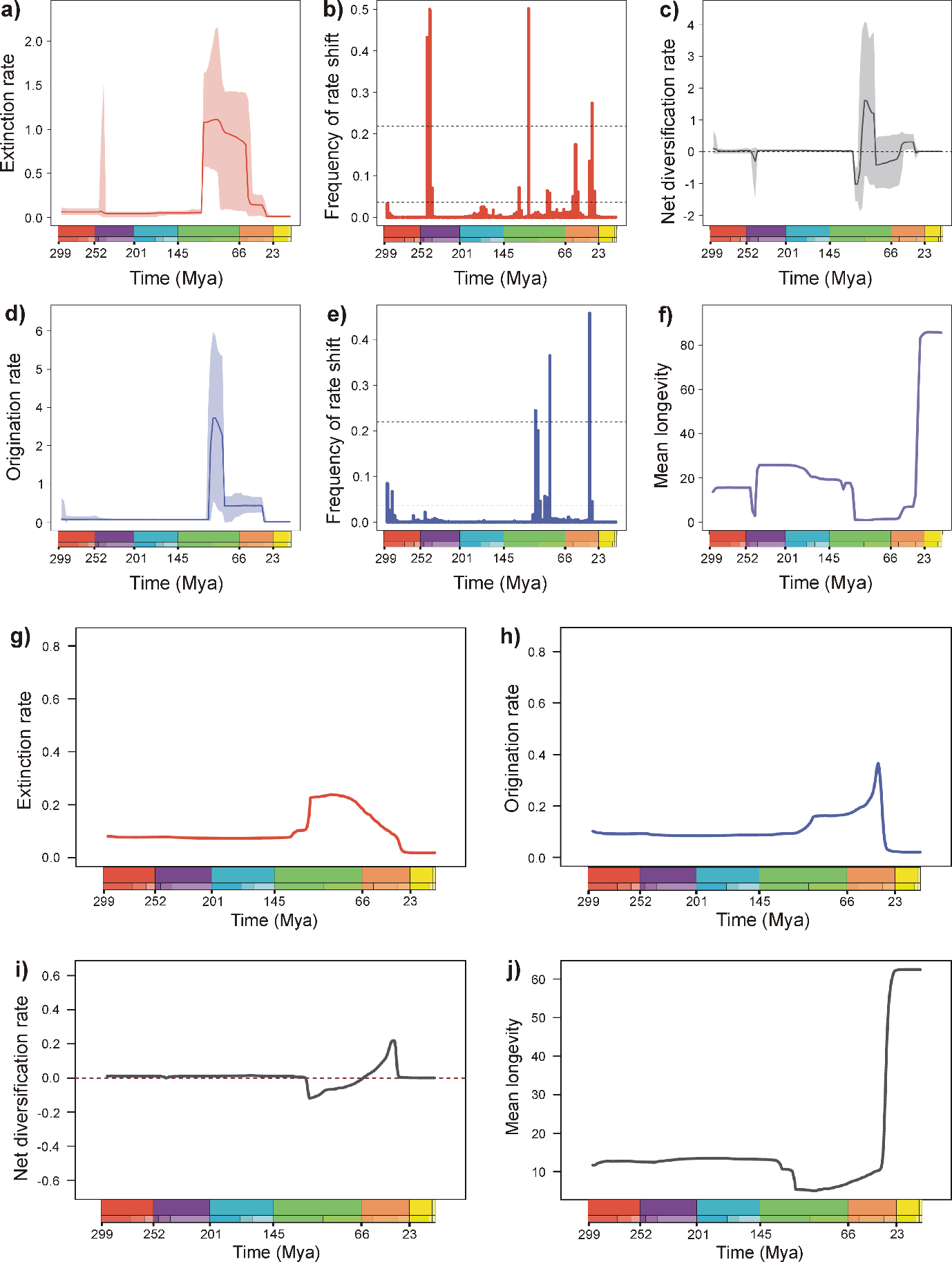

Supplement: Supplementary file 1 — Supplementary Figure 1. [file 41598_2023_42571_MOESM1_ESM.docx]
