# Supplementary material for: Fluctuation in the diversity of mayflies (Insecta, Ephemerida) as documented in the fossil record
Source: Sci Rep. 2023 Sep 25;13:16052. doi: 10.1038/s41598-023-42571-7 (PMC10519997; doi:10.1038/s41598-023-42571-7)
Supplement: Supplementary file 2 — Supplementary Table 1. [file 41598_2023_42571_MOESM2_ESM.docx]

**Supplementary Table 1.** List of mayfly fossil taxa included in the dataset.

| **Family, genus** | **Status** | **Min age** | **Max age** | **Number of occurrences** | **Species included** |
| --- | --- | --- | --- | --- | --- |
| **Acanthometropodidae** |  |  |  |  |  |
| *Analetris* | extant | 33.9 | 38.0 | 1 | *Analetris secundus* Godunko and Kłonowska-Olejnik, 2006 |
| **Aenigmephemeridae** |  |  |  |  |  |
| *Aenigmephemera* | extinct | 157.3 | 166.1 | 1 | *Aenigmephemera demoulini* Tshernova, 1968 |
| **Ameletidae** |  |  |  |  |  |
| *Baltameletus* | extinct | 33.9 | 38.0 | 1 | *Baltameletus oligocaenicus* Demoulin, 1968 |
| *Electroletus* | extinct | 33.9 | 38.0 | 1 | *Electroletus soldani* Godunko and Neumann, 2006 |
| **Ameletopsidae** |  |  |  |  |  |
| *Balticophlebia* | extinct | 33.9 | 38.0 | 1 | *Balticophlebia hennigi* Demoulin, 1968 |
| *Promirara* | extinct | 112.03 | 122.46 | 13 | *Promirara cephalota* Jell and Duncan, 1986 |
| **Ametropidae** |  |  |  |  |  |
| *Brevitibia* | extinct | 33.9 | 38.0 | 1 | *Brevitibia intricans* Demoulin, 1968 |
| *Palaeometropus* | extinct | 89.8 | 93.9 | 1 | *Palaeometropus cassus* Sinitshenkova, 2000 |
| **Australiephemeridae** |  |  |  |  |  |
| *Australiphemera* | extinct | 112.03 | 122.46 | 2 | *Australiphemera revelata* McCafferty, 1990 |
| *Borephemera* | extinct | 89.8 | 93.9 | 1 | *Borephemera goldmani* Sinitshenkova, 2000 |
| *Microphemera* | extinct | 112.03 | 122.46 | 1 | *Microphemera neotropica* McCafferty, 1990 |
| *Nanophemera* | extinct | 93.5 | 99.6 | 1 | *Nanophemera myanmarensis* McCafferty and Santiago-Blay, 2008 |
| *Palaeoanthus* | extinct | 83.5 | 85.5 | 10 | *Palaeoanthus minutus* Kluge, 1993 |
|  |  |  |  |  | *Palaeoanthus orthostylus* Kluge, 1993 |
| **Babidae** |  |  |  |  |  |
| *Baba* | extinct | 33.9 | 38.0 | 1 | *Baba lapidea* Kluge et al., 2006 |
| **Baetidae** |  |  |  |  |  |
| *Americabaetis* | extant | 13.82 | 20.44 | 7 | *Americabaetis* spp.* |
| *Cloeodes* | extant | 13.82 | 20.44 | 5 | *Cloeodes* spp.* |
| *Myanmarella* | extinct | 93.5 | 99.6 | 1 | *Myanmarella rossi* Sinitshenkova (2000) |
| *Palaeocloeon* | extinct | 83.5 | 85.8 | 9 | *Palaeocloeon taimyricum* Kluge, 1997 |
| *Vetuformosa* | extinct | 93.5 | 99.6 | 1 | *Vetuformosa buckleyi* Poinar, 2011 |
| **Baetiscidae** |  |  |  |  |  |
| *Balticobaetisca* | extinct | 33.9 | 38.0 | 4 | *Balticobaetisca stuttgardia* Godunko and Krzemiński, 2009 |
|  |  |  |  |  | *Balticobaetisca velteni* Staniczek and Bechly, 2002 |
|  |  |  |  |  | *Balticobaetisca bispinata* Staniczek et al., 2022 |
| *Protobaetisca* | extinct | 112.03 | 122.46 | 1 | *Protobaetisca bechlyi* Staniczek, 2007 |
| **Caenidae** |  |  |  |  |  |
| *Caenis* | extant | 13.82 | 20.44 | 4 | *Caenis* sp.* |
| **Coloburiscidae** |  |  |  |  |  |
| *Cronicus* | extinct | 33.9 | 38.0 | 9 | *Cronicus anomalus* (Pictet, 1854) |
|  |  |  |  |  | *Cronicus major* Demoulin, 1968 |
| *Siphlurites* | extinct | 33.9 | 38.0 | 1 | *Siphlurites explanatus* Cockerell, 1923 |
| **Cretereismatidae** |  |  |  |  |  |
| *Cretereisma* | extinct | 112.03 | 122.46 | 4 | *Cretereisma antiqua* Willmann, 2007 |
|  |  |  |  |  | *Cretereisma schwickertorum* Willmann, 2007 |
|  |  |  |  |  | *Cretereisma* spp.* |
| **Cretomitarcyidae** |  |  |  |  |  |
| *Cretomitarcys* | extinct | 89.8 | 93.9 | 1 | *Cretomitarcys luzzii* Sinitshenkova, 2000 |
| **Epeoromimidae** |  |  |  |  |  |
| *Epeoromimus* | extinct | 125.0 | 182.7 | 101 | *Epeoromimus cretaceus* Sinitshenkova, 1976 |
|  |  |  |  |  | *Epeoromimus infractus* Sinitshenkova, 1989 |
|  |  |  |  |  | *Epeoromimus kazlauskasi* Tshernova, 1969 |
|  |  |  |  |  | *Epeoromimus tertius* Tshernova, 1969 |
|  |  |  |  |  | *Epeoromimus umbratus* Sinitshenkova, 2002 |
| *Foliomimus* | extinct | 170.3 | 182.7 | 2 | *Foliomimus imitans* Sinitshenkova, 1985 |
| **Ephemerellidae** |  |  |  |  |  |
| *Clephemera* | extinct | 168.3 | 170.3 | 1 | *Clephemera clava* Lin, 1986 |
| *Ephemerella* | extant | 33.9 | 38.0 | 2 | *Ephemerella trigonoptera* Staniczek et al., 2017 |
|  |  |  |  |  | *Ephemerella* sp.* |
| *Eurylophella* | extant | 33.9 | 38.0 | 1 | *Eurylophella viscata* (Demoulin, 1968) |
| *Philolimnias* | extinct | 47.8 | 56.0 | 1 | *Philolimnias sinica* Hong, 1979 |
| *Teloganella* | extinct | 56.0 | 59.2 | 1 | *Teloganella gurhaensis* Agnihotri et al., 2020 |
| *Turfanerella* | extinct | 145.0 | 163.5 | 1 | *Turfanerella tingi* (Ping, 1935) |
| **Ephemeridae** |  |  |  |  |  |
| *Cratonympha* | extinct | 112.03 | 122.46 | 1 | *Cratonympha microcelata* Martins-Neto and Caldas, 1990 |
| *Denina* | extinct | 33.9 | 38.0 | 1 | *Denina dubiloca* McCafferty, 1987 |
| *Ephemera* | extant | 11.608 | 12.7 | 1 | *Ephemera oeningensis* Heer, 1865 |
| *Litobrancha* | extinct | 33.9 | 38.0 | 1 | *Litobrancha palearctica* McCafferty and Sinitshenkova, 1983 |
| **Euthyplociidae** |  |  |  |  |  |
| *Pristiplocia* | extinct | 112.03 | 122.46 | 22 | *Pristiplocia rupestris* McCafferty, 1990 |
|  |  |  |  |  | *Pristiplocia* sp.* |
| **Fuyoidae** |  |  |  |  |  |
| *Fuyous* | extinct | 157.3 | 163.5 | 6 | *Fuyous gregarius* Zhang and Kluge, 2007 |
| **Heptageniidae** |  |  |  |  |  |
| *Amerogenia* | extinct | 89.8 | 93.9 | 2 | *Amerogenia macrops* Sinitshenkova, 2000 |
| *Burshtynogena* | extinct | 33.9 | 38.0 | 1 | *Burshtynogena fereci* Godunko and Sontag, 2004 |
| *Ecdyonurus* | extant | 33.9 | 38.0 | 2 | *Ecdyonurus* (*Nestormeus*) *groehnorum* Godunko, 2007 |
|  |  |  |  |  | *Ecdyonurus* (*Nestormeus*) *leopoliensis* Godunko, 2004 |
| *Electrogenia* | extinct | 33.9 | 38.0 | 1 | *Electrogenia dewalschei* Demoulin, 1956 |
| *Heptagenia* | extant | 33.9 | 38.0 | 7 | *Heptagenia gleissi* Demoulin, 1968 |
|  |  |  |  |  | *Heptagenia ligata* Demoulin, 1968 |
|  |  |  |  |  | *Heptagenia senex* Demoulin, 1968 |
|  |  |  |  |  | *Heptagenia* (*Kageronia*) *fuscogrisea* (Retzius, 1783) |
|  |  |  |  |  | *Heptagenia atypica* Demoulin, 1968 |
|  |  |  |  |  | *Heptagenia bachofeni* Demoulin, 1968 |
| *Maccaffertium* | extant | 15.97 | 23.03 | 2 | *Maccaffertium annae* Macadam and Ross, 2016 |
| *Miocoenogenia* | extinct | 11.608 | 15.97 | 1 | *Miocoenogenia gorbunovi* Tshernova, 1962 |
| *Pseudokageronia* | extinct | 5.333 | 7.246 | 4 | *Pseudokageronia thomasi* Masselot and Nel, 1999 |
| *Rhithrogena* | extant | 33.9 | 38.0 | 1 | *Rhithrogena sepulta* Demoulin, 1968 |
| *Succinogenia* | extinct | 33.9 | 38.0 | 1 | *Succinogenia larssoni* Demoulin, 1965 |
| **Hexagenitidae** |  |  |  |  |  |
| *Baikalogenites* | extinct | 113.0 | 125.0 | 9 | *Baikalogenites firmus* Sinitshenkova, 2017 |
| *Caenoephemera* | extinct | 125.45 | 130.0 | 1 | *Caenoephemera shangyuanensis* Lin and Huang, 2001 |
| *Costalimella* | extinct | 112.03 | 122.46 | 2 | *Costalimella nordestina* Martins-Neto, 1996 |
|  |  |  |  |  | *Costalimella zucchii* Zamboni, 2001 |
| *Cratohexagenites* | extinct | 112.03 | 122.46 | 4 | *Cratohexagenites minor* Staniczek, 2007 |
|  |  |  |  |  | *Cratohexagenites* longicercus Staniczek, 2007 |
| *Ephemeropsis* | extinct | 113.0 | 130.0 | 1215 | *Ephemeropsis martynovae* Tshernova, 1961 |
|  |  |  |  |  | *Ephemeropsis melanurus* Cockerell, 1924 |
|  |  |  |  |  | *Ephemeropsis middendorfi* (Handlirsch, 1906) |
|  |  |  |  |  | *Ephemeropsis trisetalis* Eichwald, 1864 |
| *Epicharmeropsis* | extinct | 125.45 | 130.0 | 11 | *Epicharmeropsis hexavenulosus* Huang et al., 2007 |
|  |  |  |  |  | *Epicharmeropsis quadrivenulosus* Huang et al., 2007 |
| *Hexagenites* | extinct | 145.0 | 150.8 | 5 | *Hexagenites cellulosus* (Hagen, 1862) |
| *Hexameropsis* | extinct | 93.5 | 132.9 | 6 | *Hexameropsis africana* Sinitshenkova, 1975 |
|  |  |  |  |  | *Hexameropsis selini* Tshernova and Sinitshenkova, 1974 |
|  |  |  |  |  | *Hexameropsis elongatus* Lin et al., 2018 |
| *Libanoephemera* | extinct | 125.45 | 130.0 | 1 | *Libanoephemera inopinatabranchia* Azar et al., 2019 |
| *Mongologenites* | extinct | 113.0 | 125.0 | 774 | *Mongologenites laqueatus* Sinitshenkova, 1986 |
| *Protoligoneuria* | extinct | 112.03 | 122.46 | 437 | *Protoligoneuria limai* Demoulin, 1955 |
|  |  |  |  |  | *Protoligoneuria heloisae* Storari et al., 2022 |
| *Shantous* | extinct | 157.3 | 163.5 | 7 | *Shantous lacustris* Zhang and Kluge, 2007 |
| *Siberiogenites* | extinct | 125.0 | 168.3 | 42 | *Siberiogenites angustatus* Sinitshenkova, 1985 |
|  |  |  |  |  | *Siberiogenites branchicillus* Huang et al., 2011 |
|  |  |  |  |  | *Siberiogenites medius* Sinitshenkova, 1989 |
|  |  |  |  |  | *Siberiogenites mongolicus* Sinitshenkova, 2002 |
|  |  |  |  |  | *Siberiogenites recticostalis* Sinitshenkova, 2000 |
|  |  |  |  |  | *Siberiogenites rotundatus* Sinitshenkova, 1985 |
| **Isonychiidae** |  |  |  |  |  |
| *Isonychia* | extant | 30.8 | 33.3 | 1 | *Isonychia alderensis* Lewis, 1977 |
| **Jarmilidae** |  |  |  |  |  |
| *Jarmila* | extinct | 295.5 | 298.9 | 1 | *Jarmila elongata* Demoulin 1970 |
| **Leptohyphidae** |  |  |  |  |  |
| *Leptohyphes* | extant | 15.97 | 23.03 | 1 | *Leptohyphes* sp.* |
| **Leptophlebiidae** |  |  |  |  |  |
| *Aphelophlebodes* | extinct | 7.246 | 11.608 | 1 | *Aphelophlebodes stocki* Pierce, 1945 |
| *Atalophlebia* | extant | 2.588 | 5.333 | 7 | *Atalophlebia culleni* (Etheridge and Olliff, 1890) |
| *Aureophlebia* | extinct | 89.8 | 93.9 | 1 | *Aureophlebia sinitshenkovae* Peters and Peters, 2000 |
| *Blasturophlebia* | extinct | 33.9 | 38.0 | 1 | *Blasturophlebia hirsuta* Demoulin, 1968 |
| *Borinquena* | extant | 13.82 | 20.44 | 4 | *Borinquena caeciliana* Staniczek, 2003 |
|  |  |  |  |  | *Borinquena maculata* Staniczek, 2003 |
|  |  |  |  |  | *Borinquena parva* Staniczek, 2003 |
|  |  |  |  |  | *Borinquena schawallfussi* Staniczek et al., 2017 |
| *Calliarcys* | extant | 33.9 | 38.0 | 1 | *Calliarcys antiquus* Godunko et al., 2022 |
| *Conovirilus* | extinct | 125.0 | 129.4 | 1 | *Conovirilus poinari* McCafferty, 1997 |
| *Hagenulites* | extinct | 13.82 | 20.44 | 2 | *Hagenulites hitchingsi* Staniczek, 2003 |
| *Hagenulus* | extant | 13.82 | 20.44 | 1 | *Hagenulus* (*Poecillophlebia*) sp. |
| *Kachinophlebia* | extinct | 93.5 | 99.6 | 1 | *Kachinophlebia zhouchangfai* Chen and Zheng, 2022 |
| *Leptoneta* | extinct | 125.0 | 139.8 | 3 | *Leptoneta calyptrata* Sinitshenkova, 1989 |
| *Paraleptophlebia* | extant | 33.9 | 38.0 | 7 | *Paraleptophlebia prisca* (Pictet and Hagen, 1856) |
|  |  |  |  |  | *Paraleptophlebia electra* (Kluge, 1993) |
| **Litophlebiidae** |  |  |  |  |  |
| *Litophlebia* | extinct | 228.0 | 237.0 | 3 | *Litophlebia optata* Riek, 1976 |
| *Triassolitophlebia* | extinct | 242.0 | 247.2 | 1 | *Triassolitophlebia palegica* Sinitshenkova and Aristov, 2015 |
| **Mesonetidae** |  |  |  |  |  |
| *Clavineta* | extinct | 125.0 | 166.1 | 17 | *Clavineta brevinodia* Huang et al., 2011 |
|  |  |  |  |  | *Clavineta cantabilis* Sinitshenkova, 1991 |
|  |  |  |  |  | *Clavineta citima* Sinitshenkova, 2000 |
|  |  |  |  |  | *Clavineta excavata* Huang et al., 2011 |
|  |  |  |  |  | *Clavineta eximia* Zhang, 2006 |
|  |  |  |  |  | *Clavineta transbaikalica* Sinitshenkova, 2000 |
| *Furvoneta* | extinct | 113.0 | 166.1 | 88 | *Furvoneta domefacta* Sinitshenkova, 1991 |
|  |  |  |  |  | *Furvoneta khasurtensis* Sinitshenkova, 2017 |
|  |  |  |  |  | *Furvoneta lata* Sinitshenkova, 1976 |
|  |  |  |  |  | *Furvoneta lucida* Sinitshenkova, 2002 |
|  |  |  |  |  | *Furvoneta relicta* Zhang, 2006 |
|  |  |  |  |  | *Furvoneta sobria* Sinitshenkova, 2002 |
|  |  |  |  |  | *Furvoneta undina* Sinitshenkova, 1976 |
| *Mesoneta* | extinct | 125.0 | 247.2 | 165 | *Mesoneta antiqua* Brauer et al., 1889 |
|  |  |  |  |  | *Mesoneta beipiaoensis* Wang, 1980 |
|  |  |  |  |  | *Mesoneta deusta* Sinitshenkova, 2000 |
|  |  |  |  |  | *Mesoneta magna* Sinitshenkova, 1985 |
|  |  |  |  |  | *Mesoneta minuta* Sinitshenkova, 2000 |
|  |  |  |  |  | *Mesoneta mongolica* Sinitshenkova, 1989 |
|  |  |  |  |  | *Mesoneta picta* Sinitshenkova, 2000 |
|  |  |  |  |  | *Mesoneta triassica* Sinitshenkova, 2000 |
|  |  |  |  |  | *Mesoneta tushilgae* Sinitshenkova, 1989 |
|  |  |  |  |  | *Mesoneta uralensis* Sharov, 1948 |
|  |  |  |  |  | *Mesoneta utriculata* Sinitshenkova, 1985 |
|  |  |  |  |  | *Mesoneta zolensis* Sinitshenkova, 1990 |
| **Mesoplectopteridae** |  |  |  |  |  |
| *Mesoplectopteron* | extinct | 242.0 | 247.2 | 11 | *Mesoplectopteron longipes* Sinitshenkova and Papier, 2005 |
| **Metretopodidae** |  |  |  |  |  |
| *Metretopus* | extant | 33.9 | 38.0 | 5 | *Metretopus trinervis* Demoulin, 1968 |
|  |  |  |  |  | *Metretopus dividus* Staniczek and Godunko, 2014 |
| *Siphloplecton* | extant | 33.9 | 38.0 | 24 | *Siphloplecton barabani* Staniczek and Godunko, 2012 |
|  |  |  |  |  | *Siphloplecton demoulini* Staniczek and Godunko, 2012 |
|  |  |  |  |  | *Siphloplecton gattolliati* Staniczek and Godunko, 2016 |
|  |  |  |  |  | *Siphloplecton hageni* Staniczek and Godunko, 2012 |
|  |  |  |  |  | *Siphloplecton jaegeri* Demoulin, 1968 |
|  |  |  |  |  | *Siphloplecton macrops*(Hagen, 1856) |
|  |  |  |  |  | *Siphloplecton picteti* Staniczek and Godunko, 2012 |
|  |  |  |  |  | *Siphloplecton sartorii* Staniczek and Godunko, 2016 |
|  |  |  |  |  | *Siphloplecton landolti* Godunko et al., 2019 |
|  |  |  |  |  | *Siphloplecton studemannae* Godunko et al., 2019 |
|  |  |  |  |  | *Siphloplecton* spp.* |
| *Xenophlebia* | extinct | 33.9 | 38.0 | 1 | *Xenophlebia aenigmatica* Demoulin, 1968 |
| **Mickoleitidae** |  |  |  |  |  |
| *Mesogenesia* | extinct | 113.0 | 163.5 | 2 | *Mesogenesia petersae* Tshernova, 1977 |
|  |  |  |  |  | *Mesogenesia* sp. |
| *Mickoleitia* | extinct | 112.03 | 122.46 | 23 | *Mickoleitia longimanus* Staniczek et al., 2011 |
| **Misthodotidae** |  |  |  |  |  |
| *Misthodotes* | extinct | 298.9 | 254.17 | 47 | *Misthodotes biguttatus* Tillyard, 1932 |
|  |  |  |  |  | *Misthodotes delicatulus* Tillyard, 1936 |
|  |  |  |  |  | *Misthodotes dubius* Sinitshenkova, 2013 |
|  |  |  |  |  | *Misthodotes edmundsi* Carpenter, 1979 |
|  |  |  |  |  | *Misthodotes obtusus* Sellards, 1907 |
|  |  |  |  |  | *Misthodotes ovalis* Tillyard, 1932 |
|  |  |  |  |  | *Misthodotes sharovi* Tshernova, 1965 |
|  |  |  |  |  | *Misthodotes stapfi* Kinzelbach and Lutz, 1984 |
|  |  |  |  |  | *Misthodotes tshernovae* Sinitshenkova and Vassilenko, 2012 |
|  |  |  |  |  | *Misthodotes visherensis* Novokshonov et al., 2002 |
|  |  |  |  |  | *Misthodotes zalesskyi* Tshernova, 1965 |
|  |  |  |  |  | *Misthodotes* sp. |
| *Triassodotes* | extinct | 252.17 | 242.0 | 24 | *Triassodotes vogesaicus* Sinitshenkova et al., 2005 |
|  |  |  |  |  | *Triassodotes rasnitsyni* Sinitshenkova et al., 2021 |
| **Neoephemeridae** |  |  |  |  |  |
| *Neoephemera* | extant | 47.8 | 56.0 | 1 | *Neoephemera antiqua* Sinitshenkova, 1999 |
| *Potamanthellus* | extant | 30.8 | 33.3 | 1 | *Potamanthellus rubiensis* Lewis, 1977 |
| **Oboriphlebiidae** |  |  |  |  |  |
| *Oboriphlebia* | extinct | 295.5 | 298.9 | 4 | *Oboriphlebia moravica* Demoulin, 1970 |
|  |  |  |  |  | *Oboriphlebia quarta* Hubbard and Kukalová-Peck, 1980 |
|  |  |  |  |  | *Oboriphlebia quinta* Hubbard and Kukalová-Peck, 1980 |
|  |  |  |  |  | *Oboriphlebia tertia* Hubbard and Kukalová-Peck, 1980 |
| **Oligoneuriidae** |  |  |  |  |  |
| *Colocrus* | extinct | 112.03 | 122.46 | 8 | *Colocrus indivicum* McCafferty, 1990 |
|  |  |  |  |  | *Colocrus magnum* Staniczek, 2007 |
| *Incogemina* | extinct | 112.03 | 122.46 | 1 | *Incogemina nubila* Storari et al., 2020 |
| **Palingeniidae** |  |  |  |  |  |
| *Mesopalingea* | extinct | 125.45 | 130.0 | 19 | *Mesopalingea lerida* Whalley and Jarzembowski, 1985 |
| **Polymitarcyidae** |  |  |  |  |  |
| *Caririnympha* | extinct | 112.03 | 122.46 | 1 | *Caririnympha mandibulata* Martins-Neto and Caldas, 1990 |
| **Potamanthidae** |  |  |  |  |  |
| *Olindinella* | extinct | 112.03 | 122.46 | 1 | *Olindinella gracilis* Martins-Neto and Caldas, 1990 |
| **Protereismatidae** |  |  |  |  |  |
| *Alexandrinia* | extinct | 254.17 | 290.1 | 19 | *Alexandrinia directa* Carpenter, 1979 |
|  |  |  |  |  | Alexandrinia gigantea Sinitshenkova and Vassilenko, 2012 |
|  |  |  |  |  | *Alexandrinia ipsa* Sinitshenkova, 2013 |
|  |  |  |  |  | *Alexandrinia vitta* Sinitshenkova, 2013 |
| *Ponalex* | extinct | 254.17 | 259.9 | 1 | *Ponalex maximus* Sinitshenkova and Aristov, 2012 |
| *Protereisma* | extinct | 279.3 | 290.1 | 58 | *Protereisma americana* Demoulin, 1970 |
|  |  |  |  |  | *Protereisma arcuatum* Sellards, 1907 |
|  |  |  |  |  | *Protereisma elongatum* Sellards, 1907 |
|  |  |  |  |  | *Protereisma latum* Sellards, 1907 |
|  |  |  |  |  | *Protereisma permianum* Sellards, 1907 |
|  |  |  |  |  | *Protereisma insigne* Tillyard, 1932 |
| **Prosopistomatidae** |  |  |  |  |  |
| *Proximicorneus* | extinct | 93.5 | 99.6 | 2 | *Proximicorneus rectivenius* Lin et al., 2017 |
|  |  |  |  |  | *Proximicorneus* sp.* |
| **Sharephemeridae** |  |  |  |  |  |
| *Hammephemera* | extinct | 252.17 | 242.0 | 5 | *Hammephemera pulchra* Sinitshenkova, 2012 |
| *Jurassephemera* | extinct | 168.3 | 170.3 | 2 | *Jurassephemera zhangi* Zhang, 2022 |
| *Sharephemera* | extinct | 145.0 | 152.1 | 1 | *Sharephemera cubitalis*, Sinitshenkova 2002 |
| *Tunephemera* | extinct | 247.2 | 254.17 | 1 | *Tunephemera tungussica* Sinitshenkova, 2013 |
| **Siphlonuridae** |  |  |  |  |  |
| *Albisca* | extinct | 125.0 | 139.8 | 11 | *Albisca tracheata* Sinitshenkova, 1989 |
| *Australurus* | extinct | 112.03 | 122.46 | 122 | *Australurus plexus* Jell and Duncan, 1986 |
| *Bolbonyx* | extinct | 145.0 | 152.1 | 25 | *Bolbonyx ludibriosus* Sinitshenkova, 1990 |
| *Cretoneta* | extinct | 83.5 | 85.8 | 37 | *Cretoneta acmoptera* Kluge, 1993 |
|  |  |  |  |  | *Cretoneta zherichini* Tshernova, 1971 |
|  |  |  |  |  | *Cretoneta* spp.* |
| *Cheirolgisca* | extinct | 157.3 | 163.5 | 2 | *Cheirolgisca ningchengensis* Lin and Huang, 2008 |
| *Dulcimanna* | extinct | 112.03 | 122.46 | 3 | *Dulcimanna sculptor* Jell and Duncan, 1986 |
| *Mesobaetis* | extinct | 113.0 | 228.0 | 1069 | *Mesobaetis latifilamentacea* Zhang, 2006 |
|  |  |  |  |  | *Mesobaetis mandalensis* Sinitshenkova, 1989 |
|  |  |  |  |  | *Mesobaetis ornata* Sinitshenkova, 2000 |
|  |  |  |  |  | *Mesobaetis allata* Sinitshenkova, 1985 |
|  |  |  |  |  | *Mesobaetis amplectus* Sinitshenkova, 2000 |
|  |  |  |  |  | *Mesobaetis crispa* Sinitshenkova, 2017 |
|  |  |  |  |  | *Mesobaetis maculata* Hong, 1995 |
|  |  |  |  |  | *Mesobaetis sanjianfangensis* Hong, 1995 |
|  |  |  |  |  | *Mesobaetis sibirica* Brauer et al., 1889 |
| *Mogzonurella* | extinct | 145.0 | 163.5 | 10 | *Mogzonurella colorata* Sinitshenkova, 1990 |
|  |  |  |  |  | *Mogzonurella dissimilis* Sinitshenkova, 1985 |
|  |  |  |  |  | *Mogzonurella longa* Sinitshenkova, 1985 |
| *Mogzonurus* | extinct | 152.1 | 163.5 | 1 | *Mogzonurus elevatus* Sinitshenkova, 1985 |
| *Multiramificans* | extinct | 157.3 | 163.5 | 1 | *Multiramificans ovalis*, Huang et al., 2007 |
| *Olgisca* | extinct | 145.0 | 163.5 | 3 | *Olgisca angusticubitis* Lin and Huang, 2008 |
|  |  |  |  |  | *Olgisca schwertschlageri* (Handlirsch, 1906) |
| *Proameletus* | extinct | 113.0 | 152.1 | 1198 | *Proameletus branchiatus* Sinitshenkova, 2017 |
|  |  |  |  |  | *Proameletus caudatus* Sinitshenkova, 1976 |
| *Siphangarus* | extinct | 125.0 | 129.4 | 12 | *Siphangarus rotundus* Sinitshenkova, 2000 |
| *Siphlonurus* | extant | 33.9 | 38.0 | 1 | *Siphlonurus dubiosus* Demoulin, 1968 |
| **Siphluriscidae** |  |  |  |  |  |
| *Jurassonurus* | extinct | 157.3 | 163.5 | 29 | *Jurassonurus amoenus* Huang et al., 2008 |
| *Stackelbergisca* | extinct | 125.0 | 163.5 | 38 | *Stackelbergisca clara* Sinitshenkova, 2000 |
|  |  |  |  |  | *Stackelbergisca cylindrata* Zhang, 2006 |
|  |  |  |  |  | *Stackelbergisca shaburensis* Sinitshenkova, 1991 |
|  |  |  |  |  | *Stackelbergisca sibirica* Tshernova, 1967 |
| **Tintorinidae** |  |  |  |  |  |
| *Tintorina* | extinct | 237.0 | 247.2 | 2 | *Tintorina meridensis* Krzemiński and Lombardo, 2001 |
| **Torephemeridae** |  |  |  |  |  |
| *Archaeobehningia* | extinct | 152.1 | 247.2 | 2 | *Archaeobehningia edmundsi* Tshernova, 1977 |
|  |  |  |  |  | *Archaeobehningia mogutshevae* Sinitshenkova, 2000 |
| *Torephemera* | extinct | 33.9 | 38.0 | 1 | *Torephemera longipes* Sinitshenkova, 1989 |
| **Toxodotidae** |  |  |  |  |  |
| *Toxodotes* | extinct | 242.0 | 247.2 | 2 | *Toxodotes coloratus* Sinitshenkova and Marchal-Papier, 2005 |
| **Triassoephemeridae** |  |  |  |  |  |
| *Triassoephemera* | extinct | 242.0 | 247.2 | 17 | *Triassoephemera punctata* Sinitshenkova and Papier, 2005 |
| **Triassomanthidae** |  |  |  |  |  |
| *Triassomanthus* | extinct | 242.0 | 247.2 | 35 | *Triassomanthus parvulus* Sinitshenkova and Papier, 2005 |
| **Vogesonymphidae** |  |  |  |  |  |
| *Khungtukunia* | extinct | 247.2 | 251.3 | 50 | *Khungtukunia sibirica* Sinitshenkova, 2013 |
| *Palegonympha* | extinct | 242.0 | 247.2 | 1 | *Palegonympha triassica* Sinitshenkova and Aristov, 2015 |
| *Vogesonympha* | extinct | 242.0 | 247.2 | 32 | *Vogesonympha ludovici* Sinitshenkova and Papier, 2005 |
| **Voltziaephemeridae** |  |  |  |  |  |
| *Voltziaephemera* | extinct | 242.0 | 247.2 | 251 | *Voltziaephemera fossoria* Sinitshenkova and Papier, 2005 |
| **Uncertain family** |  |  |  |  |  |
| *Astreoptera* | extinct | 112.03 | 122.46 | 1 | *Astreoptera cretacica* Brandao et al., 2021 |
| *Durlophlebia* | extinct | 139.8 | 145.0 | 1 | *Durlophlebia radleyi* Sinitshenkova and Coram, 2002 |
| *Huizhougenia* | extinct | 122.46 | 125.45 | 1 | *Huizhougenia orbicularis* Lin, 1980 |
| *Minorella* | extinct | 242.0 | 247.2 | 7 | *Minorella virgata* Sinitshenkova and Papier, 2005 |
| *Parabaetis* | extinct | 41.3 | 47.8 | 1 | *Parabaetis eocaenicus* Haupt, 1956 |
| *Phthartus* | extinct | 265.1 | 190.8 | 5 | *Phthartus africanus* Haughton, 1924 |
|  |  |  |  |  | *Phthartus netschajevi* Handlirsch, 1904 |
|  |  |  |  |  | *Phthartus rossicus* Handlirsch, 1904 |
| *Schistonotorum* | extinct | 125.45 | 130.0 | 1 | *Schistonotorum wallisi* Jarzembowski and Wang, 2019 |

**Remarks:** Families and genera within families are listed alphabetically. Assignment of individual genera to families follows original authors or subsequent revisions, where available.

Taxa marked with asterisk represent formally undescribed species, investigated by us and attributable to the known genera.

Several formally described taxa were excluded from the dataset mainly because of their uncertain attribution and original specimens of poor preservation. These include: *Baetis gigantea* Hagen, 1856; *Baetis grossa* Hagen, 1856; *Cinygma baltica* Demoulin, 1968; *Cloeon emmavillensis* Riek, 1954; *Ephemera exsucca* Scudder, 1890; *Heptagenia fluminea* Zhang, 1989; *Ephemera howarthi* Cockerell, 1908; *Ephemera immobilis* Scudder, 1890; *Ephemera interempta* Scudder, 1890; *Ephemera macilenta* Scudder, 1890; *Ephemera pumicosa* Scudder, 1890; *Ephemera tabifica* Scudder, 1890; *Heptagenia shanwangensis* (Hong 1983); *Lepismophlebia platymera* (Scudder, 1890); *Metretopus henningseni* Demoulin, 1965; *Protereisma apicalis*Martynov, 1928; *Protereisma rossenrayensis*Guthörl, 1965; *Protereisma sellardsi*Tillyard, 1932; *Protereisma uralicum* Zalessky, 1946.

The genus *Teloganella* was treated as extinct, since the fossil record of this genus does not represent extant *Teloganella*, but a related undescribed fossil genus (unpublished data).
